# Public health implications of increasing trends in the burden of road traffic accidents in Ho and Hohoe Municipalities in the Volta Region of Ghana

**DOI:** 10.1371/journal.pgph.0003238

**Published:** 2024-06-27

**Authors:** Pious Afrane, Robert Kaba Alhassan, Maxwell Afetor, Martin Adjuik Alhassan, Paul Amuna, Seth Owusu-Agyei

**Affiliations:** 1 Fred Newton Binka School of Public Health, University of Health and Allied Sciences, Hohoe, Ghana; 2 Institute of Health Research, Centre for Health Policy and Implementation Research, University of Health and Allied Sciences, Ho, Ghana; 3 University of Dundee, Scotland, United Kingdom; University of Chicago, UNITED STATES

## Abstract

Road traffic accidents account for 1.35 million deaths and up to 50 million injuries each year globally, mostly among persons aged 5–29 years. The existing road safety measures in Ghana are grossly inadequate, leading to occurrence of unprecedented Road Traffic Accidents (RTA). This study sought to document the epidemiological indices and determine the public health implications of Road Traffic Accidents and the socio-economic effect on lives of RTA victims in Ho and Hohoe Municipalities of the Volta Region in Ghana. A cross-sectional study was carried out among 198 road traffic accident victims who reported to three health facilities in the Volta Region of Ghana. The victims were interviewed using a structured questionnaire. Principal component analysis was done to categorize RTA victims into various economic status. The results show that out of the 198 road traffic accident victims who were interviewed, 50% were breadwinners of their families. Approximately 40% of the accident cases happened between 12 mid-day and 6 o’clock in the evening. Among the RTA cases recorded, 35% involved four-wheeler vehicles. Majority (88%) of those with injuries resulting from motorcycle accidents sustained a head injury; 70% of the respondents who sustained a disability from RTA were unable to perform activity of daily living (i.e. bathing, oral care, and toileting). The average cost of care on RTA victims from formal and informal health care facilities, at the time of this study, was GHC 902 (US$150) and GHC 724 (US$120) respectively. In conclusion, the increase in usage of motorcycles as a means of transport has contributed significantly to the incidence of RTAs in the Ho and Hohoe municipalities posing a public health concern. Majority of RTAs resulted in head injuries and other disabilities which affected their ability to perform activities of daily living and posed significant economic cost to victims and their families who are mostly already in lower wealth quintiles.

## Background

Road traffic accident (RTA) is defined as a collision or incident involving at least one road vehicle in motion that can be on a public or private road to which the public have the right of access. RTAs constitute one of the world’s known causes of disability and a leading cause of deaths [1]. Although there is increasing concern all over the world regarding the incidence of RTAs, the problem is more rife in developing countries as RTAs continues to be on the increase with accompanied mortalities, disabilities, social and economic impact, thus posing major public health concerns [2] According to the Global Status Report on Road Safety 2018, RTA is the eighth leading cause of deaths worldwide. It accounted for about 1.35 million deaths and up to 50 million injuries each year, especially among persons aged 5–29 years [1]. Among all deaths resulting from road traffic accidents globally, pedestrians and cyclists contribute 26%, car occupants contribute 29% and unidentified road users contribute 17% [1]. It is estimated that between the period of 2015–2030, the macroeconomic loss of road injuries will be over $1.797 trillion. This translates to the burden of road injuries being equivalent to an annual tax of 0.12% on global output, with an average per capita burden of $231 [3].

Even though there are variations in death rates for road traffic accidents among the regions across the world ranging from 9.3 to 26.6 per 100,000 people, Africa has one of the highest rates of road traffic deaths in the world with a rate of 26.6 per 100,000 people [1]. In 2017, Africa recorded RTA prevalence of 11,177,501 (consisting of 6,579,810 males and 4,597,690 females) with 161,647 fatalities (comprising of 114,479 males and 46,898 females), which was an increase from previous years’ figures [4].

According to Islam et al [5], reasons like excessive speed of the vehicles, inexperienced drivers, reckless driving, violation of traffic rules and signs are commonly associated with road traffic accidents. Age and driving experience have been noticed as a risk factor for RTAs. In a study conducted in Nepal, it was established that, drivers who were in the ages less than or equal to 30 years were more likely to be exposed to an accident than those who were above 30 years. The study further indicates that those driving at a speed less than 40 km/hr were less likely to be exposed to an accident than those with speed 40–60 km/hr and those with speed more than 60 km/hr [6].

**Fig 1:**
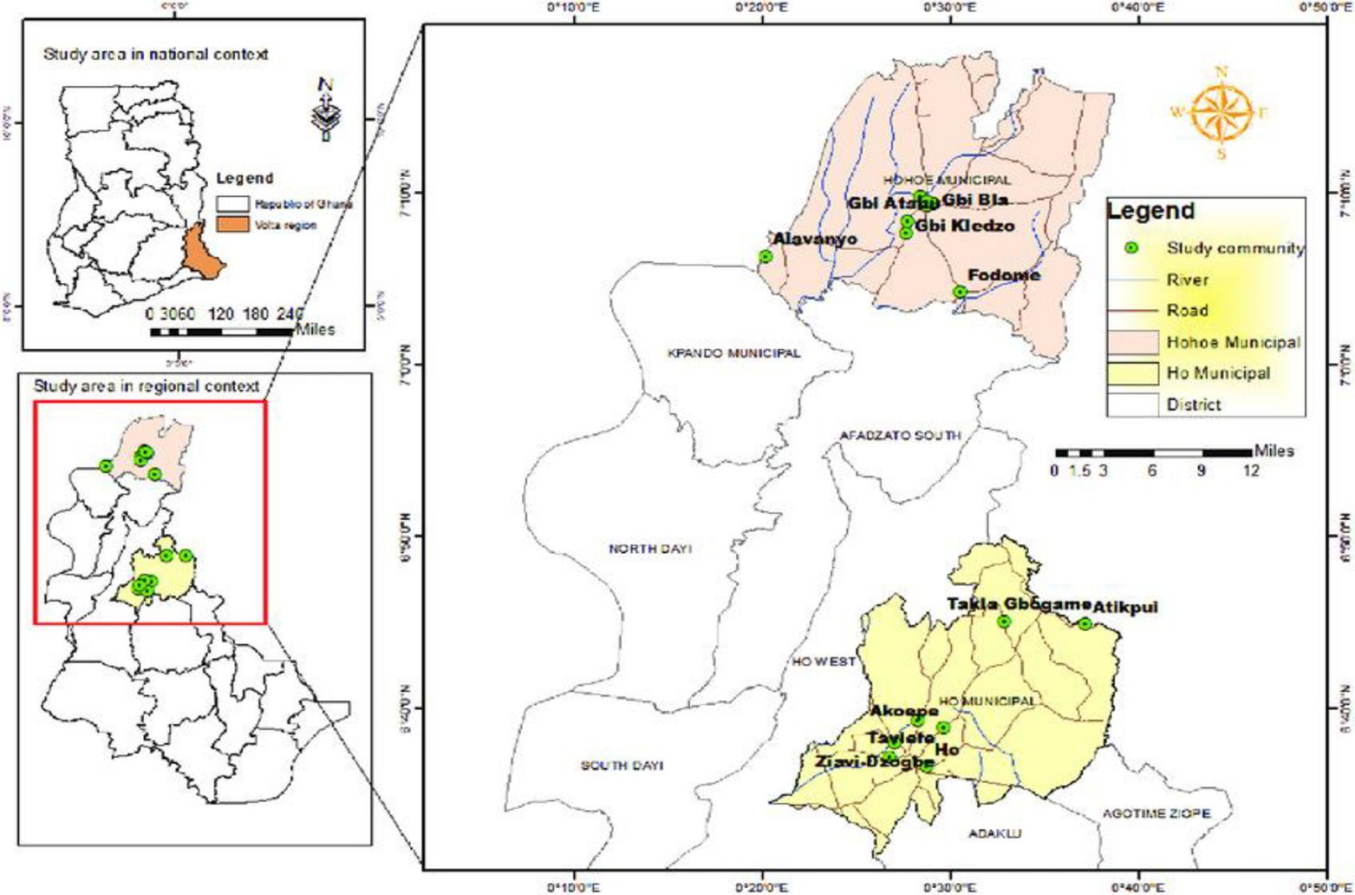
Map of Ho and Hohoe municipalities. **Source:** Morgan et al (2023) [12].

In Ghana road traffic crashes continue to cause substantial number of deaths. In 2010, there were 12,981 road accidents with 11,147 injuries and 1,760 deaths and these figures increased to 13,272 with 2,330 deaths in 2011. Increasing motorization and poor surveillance of road users, human and technical errors are among the several reasons accounting for the alarming rate of RTAs [7]. A study by Ackaah and Afukaar found that the prevalence of helmet use among motorcyclists in the Tamale Metropolis was 34.2% [8]. Similarly the use of helmet by motocylce users in the Greater Accra region were few [9]. Due to these evidences, Dapilah et al argued that road traffic accidents will continue to increase if an intensive and well cordinated road traffic education on safe traffic behaviour is not adopted in the country [10]. According to Konlan et al [11], out of 120 motorcyclists interviewed in Central Tongu in the Volta region, 63.0% of the respondents have been involved in at least one accident. This trend raises public health concern, given that, residents in the Volta Region largely depend on motorcycles as one of their major means of commercial transport due to the region’s proximity to the borders of Togo. Ho and Hohoe municipalities are among the most commercial cities in the Volta region with greater population densities hence higher motorisation. The two municipalities also share borders with Togo, as such the use of motorcycles are prominent (see Fig 1). These among other factors, such as poor road infrastructure could be a contributing factor to the higher incidence of RTAs in these municipalities.

To develop strategies to curb the menace, the cases, causes, and underlying factors have to be determined, documented and studied to guide steps to be taken to curb future occurrence of RTAs. This study sought to assess the impact of RTA on the social and economic lives of the victims in Ho and Hohoe Municipalities.

## Methods

This study employed descriptive cross-sectional design aimed at ascertaining the effects of RTAs on the social and economic lives of RTA victims in the Ho and Hohoe Municipalities of the Volta Region of Ghana. Victims of RTAs were interviewed using a structured questionnaire to ascertain the demographic characteristics, history of RTAs, cost incurred in treatment, nature of the commutable road, type of vehicle involved in the accidents they were involved in, part of body that was injured, disability status, job loss.

### Ethics approval and consent to participate

This study protocols and methods were approved by the Research and Ethics Committee of the University of Health and Allied Sciences in Ghana (UHAS-REC A.9 [117] 20–21). Each participant provided informed consent prior to each interview. Participation in the study was voluntary. All methods were carried out according to standard guidelines and regulations.

### Sample

Census was done to recruit all injuries that reported to Ho teaching Hospital, Hohoe Municipal Hospital and Ho Municipal Hospital for the period, January 2020–December 2021. Injuries that were not related to RTAs were excluded. RTAs that did not occur within the study areas were also excluded. A total of 198 injury participants were eventually interviewed to assess the type of injuries (morbidities) victims sustained after RTA as well as identify the effect of RTAs on their social and economic lives.

### Data collection methods

A total of 198 RTA victims participated in the study. A list of all the cases was obtained from the hospitals’ records department based on inpatients, outpatients and those returning for review. The patients discharged were traced to their current homes/locations. The interviews took place between January and March 2022. Patients who had been discharged were traced to their homes for interview, some patients were interviewed on phone on rare occasions where it was impossible to visit them at home. Outpatients RTA cases with minor injuries were administered with questionnaires in a private room after receiving medical care and consenting to participate in the study. Inpatients in their various wards were administered with the questionnaires if eligible and able to respond. Relatives of seriously injured patients were interviewed. Questionnaire covered demographic characteristics, history of RTAs, cost incurred in treatment, employment and job security, nature of the commutable road, type of vehicle in which he/her had accident, type of injury sustained, disability status and job loss.

### Data analysis

Data collected from questionnaire administration were checked for completeness and then entered into EpiData version 4.60 and then exported into STATA 16 for analysis. Continuous data such as age and cost of treatment were presented as means and standard deviations. Categorical variables were presented as frequencyy and proportion. Results were then presented in tables and charts. Determinants of RTAs were also presented as categorical variables. Principal Component analysis was used to categorize RTA victims into poor, moderate and rich.

## Results

### Demographic characteristics of road traffic accident victims

As shown in Table 1, a total of one hundred and ninety-eight (198) respondents of road traffic accident victims from Ho and Hohoe Municipalities were interviewed using a structured questionnaire. The age distribution of respondents showed 8.6% were below the age of 20 years, 30.3% were between the ages of 20–30 years, 38.4% were between ages 31and 40years with 22.7% above 40 years.

**Table 1:** Demographic characteristics of road traffic accident victims (n = 198).

Variable		Frequency	Percentage
**Age group**			
	less than 20	17	8.6
	20–30	60	30.3
	31–40	76	38.4
	40 and above	45	22.7
**Sex**			
	Male	146	73.7
	Female	52	26.3
**Religion**			
	Christianity	177	89.4
	Muslim	16	8.1
	Traditionalist	1	0.5
	Others	4	2
**Level of education**			
	JHS	63	31.8
	None	3	1.5
	Primary	14	7.1
	SHS	71	35.9
	Tertiary	47	23.7
**Bread winner**			
	No	100	50.5
	Yes	98	49.5
**Time of day Accident occurred**			
	12am-6am	11	5.6
	12pm-6pm	80	40.4
	6am-12pm	43	21.7
	6pm-12am	64	32.3

Source: Field Data (2022)

The sex distribution showed that, 146 (73.7%) were males and 52 respondents (26.3%) were females. In relation to their educational levels, 1.5% did not have any formal education, 7.1% had primary education, 31.8% had JHS education, 35.9% had their secondary education and 23.7% had their tertiary education. A split of 49.5% of the respondents were bread winners of their families whilst 50.5% were not bread winners. Only 5.6% of all accidents happened between the hours of 12 midnight and 6 am, compared to 21.7% between 6 am and 12 middays, 40.4% between 12 midday and 6 pm and 32.3% in the hours of 6 pm and12 midnight.

### Subjective factors that resulted in the various RTAs

**Fig 2:**
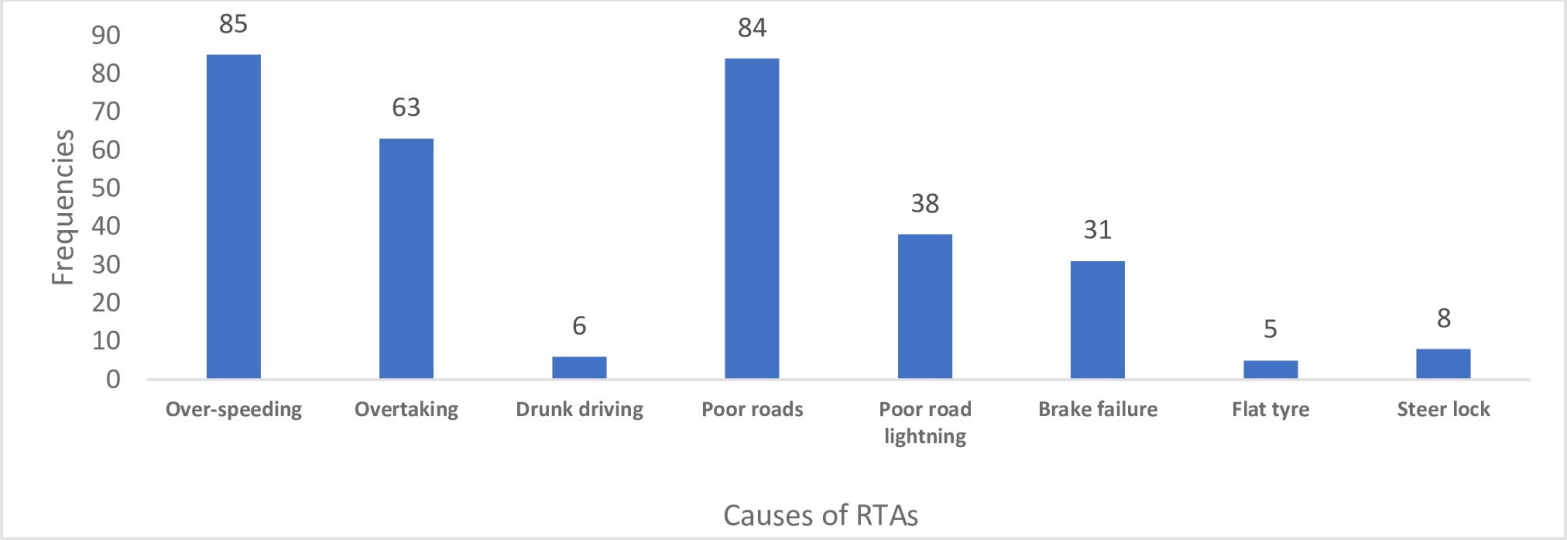
Subjective factors that resulted in the various RTAs. **Data source:** Field data (2022).

The results indicate that, 43.2% of the respondents mentioned that their accidents occurred as a result of over-speeding, 42.4% attributed their accident to poor roads, 31.8% mentioned their accident was as a result of overtaking, 19.2% mentioned poor road lighting, 15.7% said their accident was due failed brakes of vehicle, 4.0% mentioned they had accident due to steering lock, 3.0% mentioned the driver was drunk, and 2.5% attributed their accident to a tyre burst. Thus, 154 respondents attributed human behaviour (over speeding, overtaking, drunk driving) to the cause of their accidents, whilst 122 of the respondents attributed their accidents to poor road related cause (poor road, poor road lighting) and 44 attributed it to vehicular-related cause (brake failure, tyre burst and steering lock) (see Fig 2).

### Types of vehicles contributing to RTAs in Ho and Hohoe municipalities

**Fig 3:**
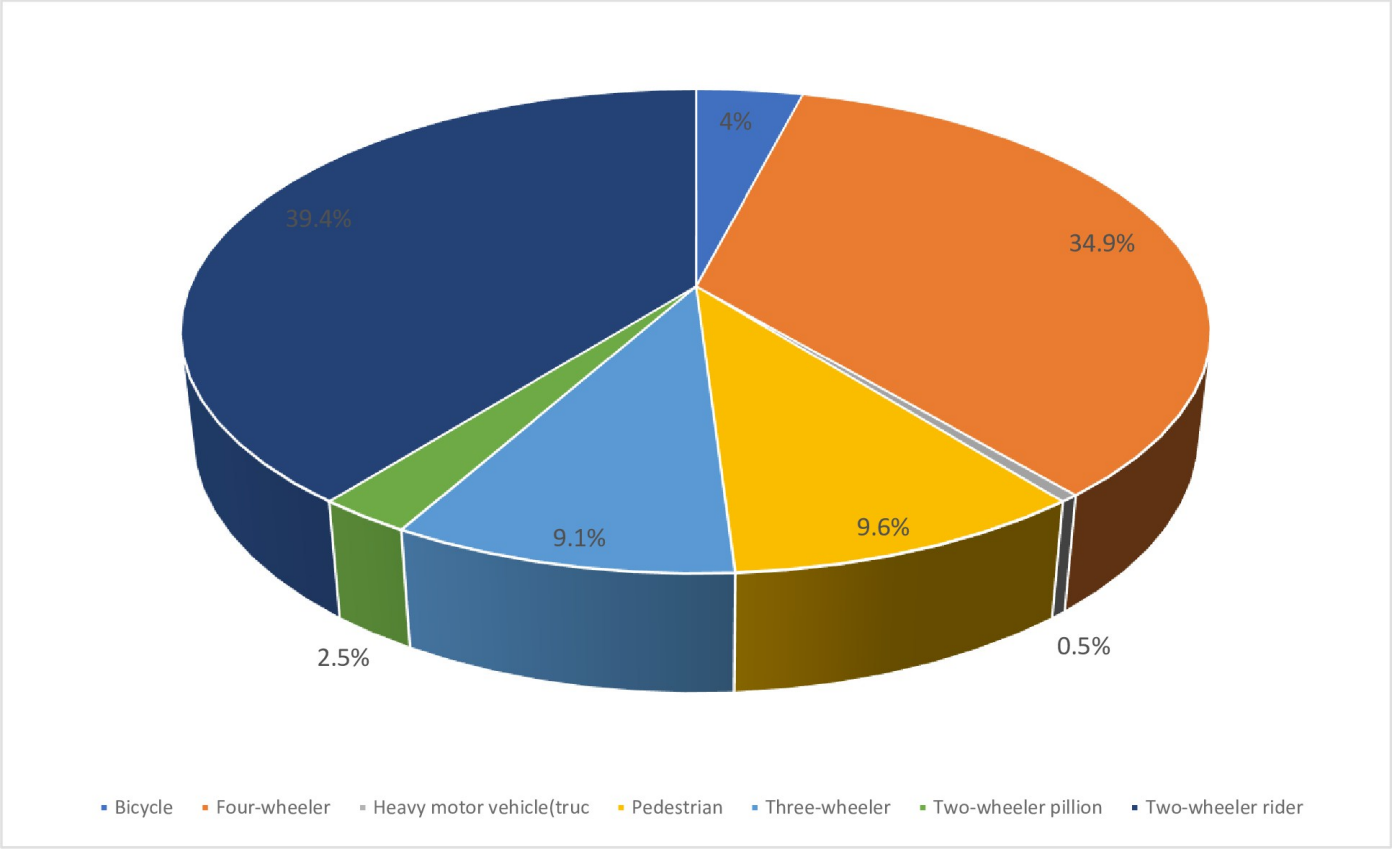
Types of vehicles contributing to RTAs in Ho and Hohoe municipalities, 2022. **Data source:** Field data (2022).

Fig 2 shows that, among all cases of RTAs 4.0% were bicycles, 34.9% were four-wheeler vehicles, 0.5% were Heavy motor vehicles(trucks), 9.6% were pedestrians, 9.1% were three-wheeler, 2.5% were two-wheeler pillion motor and 39.4% were two-wheeler motorcycles. All motorcycles combined (three-wheeler, two-wheeler pillion, two-wheeler rider) were as high as 51.0% of all RTA cases (see Fig 3).

### Injury characteristics of RTA victims in Ho and Hohoe municipalities

Table 2 indicates that, for all head injuries, 39.4% were abrasions, 14.4% were contusions, 41.7% were lacerations and 5.6% were fractures; for all Thoracic-Abdominal Injury, 26.7% were abrasions, 13.3% were contusions, 32.2% were lacerations and 3.3% were fractures. Also, for all Injury of the extremities, 43.8% were abrasions, 15.0% were contusions, 42.8% were lacerations and 41.2% were fractures. For all Injury to the Spine 7.6% were abrasions, 2.5% were contusions, 8.6% were lacerations and 0.5% were fractures.

**Table 2:** Characteristics of injuries associated with RTA victims in Ho and Hohoe municipalities.

Variables		Frequency	Percentage %
* **Head Injury**			
	Abrasion	71	39.4
	Contusion	26	14.4
	Laceration	75	41.7
	Fractures	10	5.6
**Injury of the Extremities**			
	Abrasion	85	43.8
	Contusion	29	15.0
	Laceration	83	42.8
	Fractures	80	41.2
* **Injury to the Spine**			
	Abrasion	15	7.6
	Contusion	5	2.5
	Laceration	17	8.6
	Fractures	1	0.5

Source: Field Data (2022)

*Multiple choice question.

### Morbidities/injuries associated with various types of vehicles causing RTAs

Table 3 shows the kind of injuries associated with various vehicle types that the victims had the accidents with. For head injuries, 65(92.9%) of all injuries resulting from car accidents had head injuries and 89(88.1%) of all injuries resulting from motorcycle accidents sustained a head injury.

**Table 3:** Morbidities/injuries associated with various types of vehicles causing RTAs in Ho and Hohoe municipals 2021.

Part of Body Injured		Type of vehicle involved in accident			
		Bicycle(n = 8)	Cars(n = 70)	Pedestrian(n = 19)	Motorcycle(n = 101)
**Head**					
	No	1(12.5)	5(7.1)	0(0)	12(11.9)
	Yes	7(87.5)	65(92.9)	19(100.0)	89(88.1)
**Abdomino-thoracic Region**					
	No	3(37.5)	31(44.3)	10(52.6)	50(49.5)
	Yes	5(62.5)	39(55.7)	9(47.4)	51(50.5)
**Extremities**					
	No	1(12.5)	5(7.1)	0(0)	11(10.9)
	Yes	7(87.5)	65(92.9)	19(100)	90(89.1)
**spine and Lumber Region**					
	No	6(75.0)	52(74.3)	18(94.7)	87(86.1)
	Yes	2(25.0)	18(25.7)	1(5.3)	14(13.9)

Source: Field Data (2022).

For abdomino-thoracic injuries, 39(55.7%) of all car accidents sustained abdomino- thoracic injury and 51(50.5%) of all motorcycle accidents sustained abdomino-thoracic injury.

For injuries on the extremities, 65(92.9%) resulting from car accidents were injuries on the extremities and 90(89.0%) of all injuries resulting from motorcycle accidents were injuries on the extremities.

Lastly, for injuries of the spine, 14(13.9%) of all injuries resulting from motorcycles sustained a spine injury and 18(25.7%) of all car accidents sustained an injury of the spine.

### Economic Characteristics of injured victims of RTA

**Fig 4:**
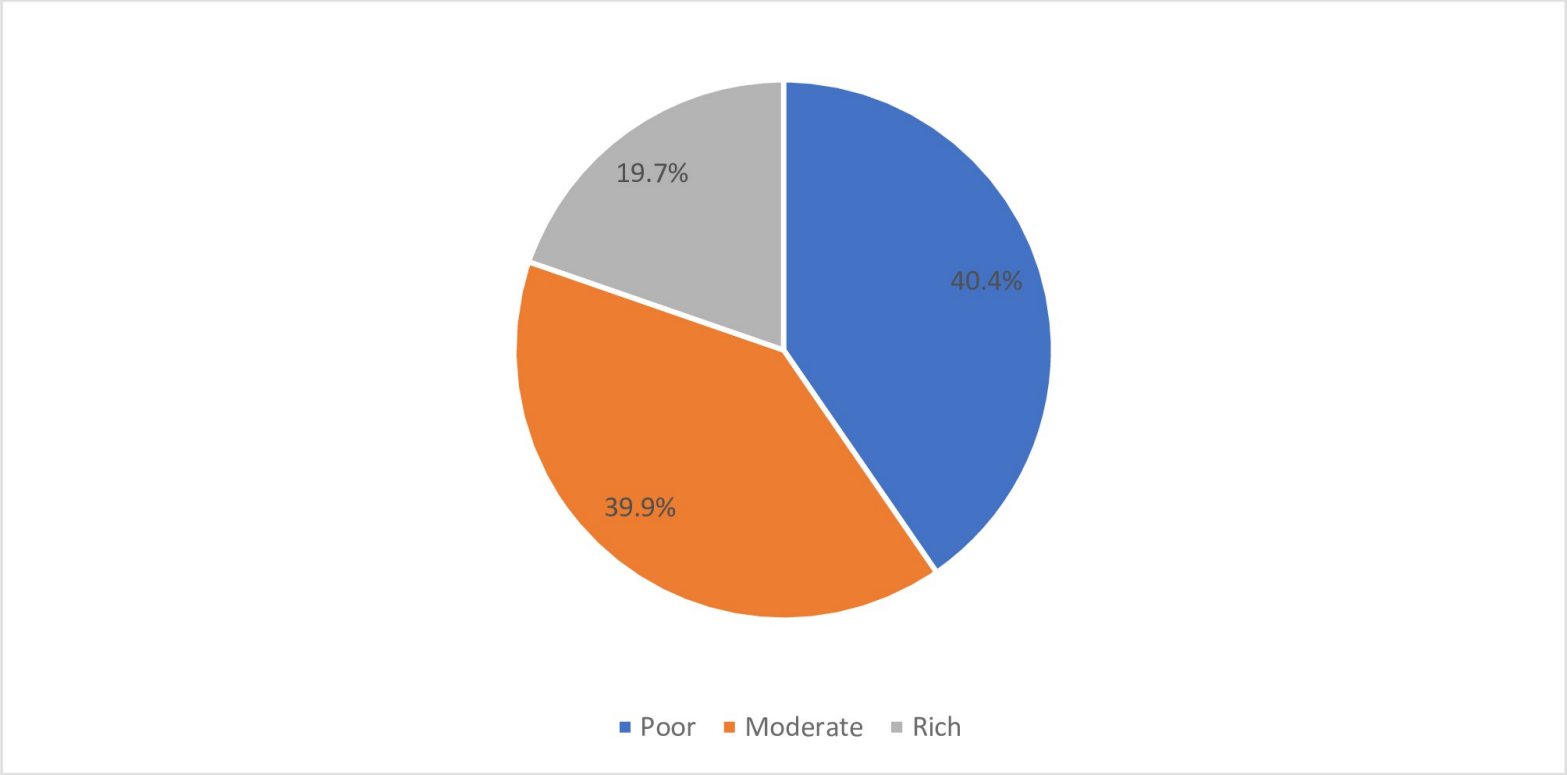
Economic status of RTA victims in Ho and Hohoe municipalities, 2022. **Data source:** Field data (2022).

Fig 3 shows 80(40.4%) of the RTA victims were poor, 79(39.9%) were moderately rich and 39(19.7%) were rich (see Fig 4).

Table 4 indicates the economic characteristics of RTA victims in Ho and Hohoe Municipalities in the Volta region of Ghana. The results indicates that,162 respondents representing 81.8% of all the respondents were working before they had the accidents, 28.3% of the respondents had a disability because of their accidents. A significant number 39(69.6%) out of the 56 respondents who sustained a disability were unable to perform activity of daily living. Close to three quarters, 74.7%, of the respondents were able to return to work after their accident whilst 25.3% were unable to return to work after their accident. As high as 113(69.8%) respondents stayed out of work for over one month. Also 22.2% of respondents lost their jobs because of their accidents and 58.6% had their earnings reduced. The average cost of care on RTAs for both formal facilities and informal were GHC 902(US$150) and GHC724 (US$120) respectively, based on an exchange rate of 1 US$ equivalent to 6.0 Ghana Cedis (GHC) at the time of conducting the study.

**Table 4:** Economic characteristics of injured victims.

Variable		Frequency	Percentage
**Sustain Disability**			
	Yes	56	28.3
	No	142	71.7
**Activity of Daily living**			
	Able	39	69.6
	Unable	17	30.4
**Working status before accident**			
	Was working	162	81.8
	Was not working	36	18.2
**Return to work after this accident**			
	Yes	121	74.7
	No	41	25.3
**Days of work lost**			
	One day	5	3.1
	2–4 days	2	1.2
	5–7 days	6	3.7
	1-2weeks	16	9.9
	3–4 weeks	20	12.3
	Over 1 month	113	69.8
**Lost Job as a result of accident**			
	Yes	36	22.2
	No	126	77.8
**Earnings reduced as a result of accident**			
	Yes	67	58.6
	No	95	41.4

Source: Field Data (2022).

## Discussion

The study results found that, majority of the road traffic accident (RTA) victims were within their productive ages of 20 and 39 years; many were males 146 (73.7%), with a fair proportion being providers for their families. This agreed with a study carried out by Mohammed et al [13] which reported that demographic factors like age and sex affect motorcycle accident occurrence. Another study conducted in the northern part of Ghana stated that majority of young adults within ages 20 and 39 years, particularly males, were the ones mostly involved in accidents [14]. Also, a previous hospital-based study carried out in Tanzania indicated that 67.2% of the road traffic accident victims were males [15].

This study findings align with the conclusions drawn in their research report, which suggests that the youth especially the males’ inclination towards adventure and willingness to take risks, such as engaging in dangerous driving may be the reason behind this consistency (Steinberg, 2008). This implies that the younger generation, who represent the future of the nation, are more prone to motorcycle accidents. Again, majority of the victims being bread winners suggest that, the nature of their work either as motor riders/car drivers or as passengers exposes them to RTAs It is therefore necessary to implement stricter measures in order to effectively address this problem.

Also, this study observed that the highest proportion of RTA victims were involved in accidents that occurred between midday (i.e. 12.00 hours) and 18.00 hours followed by competitive number of victims whose RTAs occurred in the hours of 18 hours and midnight (i.e. 24.00 hours). On the contrary, a study carried out in Iran analysed time distribution trend of accidents and observed that most of the accidents that occurred took place between 16:00 hours and 20:00 hours in the day [16]. This may be attributed to time and socioeconomic differences in geographical areas. In Ghana, the hours between 12.00 hours (midday) and 18.00 hours are the active working hours. Also, from 18.00 hours is the rush hours of commuters to their various homes, as such more vehicular and human movement occurred around these hours. This is not different from the situation of commuters in the Volta region where this study was carried out.

In this study it was observed that RTAs were more common among two-wheeler motorcycles than any other vehicle. This was consistent with the findings in a study conducted in India, which reported that RTAs were more common in two-wheel drivers followed by pedestrians [17].

However, in this study, the second common vehicles associated with RTAs was found to be the four-wheeler drives this contradicts the findings of Siddaramanna and Kumar [17], where the second common type of RTAs were associated with pedestrians. This contradiction could be because of human population difference in Ghana and India and the number of pedestrian commuters on the roads in each country. The larger the human population, the higher the pedestrian commuters and the more likely they can be knocked down by moving vehicles.

In contrast, a study on the pattern of head injuries in Road Traffic Accidents in Imphal by Slong and Devi [18], found that truck drivers were the highest vehicle offenders of RTAs. However, it was not clear how often or the rate of use of motorcycles in the study area. The higher the usage of motorcycles other than trucks, the more likely motorcycles may be involved in RTAs. In Ho and Hohoe where this study was conducted, commuters are more likely to use motorcycles other than trucks.

Describing the injuries sustained by RTA victims, this study results indicate that majority of respondents reported injuries of extremities (lower and upper limbs) followed by head injuries. One person could however, sustain injuries to more than one part of the body. Similarly, according to the article published by Mittal et al [19], on pattern of common injuries in road traffic accidents, out of 1518 medico-legal autopsies studied for deaths resulting from RTAs, musculoskeletal injuries, especially the limbs, were the highest (47.0%) among the list of RTA injuries in all age groups. In many instances, this included wounds which are in combinations of abrasion, contusion, lacerations, and fracture dislocation.

Again, this study indicated that, head injury was common among respondents who had accidents with motorcycles. This is in consistent with another study, which observed that, head injuries were common among motorised two-wheelers representing 22.6% of all cases studied [20].

In assessing the effects of RTAs on the socio-economic lives of victims in Ho and Hohoe Municipalities in the Volta Region, this study results indicated that, majority 39(69.6%) of the respondents who sustained a disability were unable to perform activity of daily living (e.g. bathing, oral care and toileting) and a number of all RTA victims were unable to return to work after their accident; the average direct cost of informal and formal treatments were relatively high.

A similar study conducted in Nigeria reported that, out of 3082 participants, 29.1% had a disability owing to RTAs; 13.5% were unable to return to work and the average direct costs of informal and formal treatment were US $6.65 and US$35.64, respectively [21]. The difference in cost of treatment may be because of the exchange rate in the two countries as at the time of the study. Also, the period difference and the difference in the cost of living in Ghana and in Nigeria might have affected cost of treatment.

This study found out that almost half (49.5%) of the RTA victims were providers for their families and almost a quarter (22.22%) of them lost their jobs because of the accidents they were involved in. Also, more than half (58.6%) had their earnings reduced. It can be inferred that RTAs significantly affect the most active and productive part of the human population, which leads the country to incur economic loss.

### Public health implication of RTAs

This study identified human factors, vehicular factors and road conditions contributed to all the accidents that the victims suffered, as majority (154) of the respondents mentioned that, human behaviour,such as over-speeding, overtaking, drunk-driving, was the cause of accidents that they were involved in; 122 of the respondents attributed their accidents to road-related cause, such as unmotorable roads, poor road lighting; the remaining 44 attributed it to vehicular related cause, brake failure, tyre burst and steer lock.

Similarly, in assessing the problem of RTAs in Ghana, Coleman in his study indicated per the National Road Safety Authority’s report in 2007 that six people were killed daily on our roads and 25% of all pedestrian deaths involved children [22]; this highlights the public health implication of RTAs, concluding that RTAs were mostly attributed to vehicular, human and road factors. Law-enforcers have, hhowever, failed to curb the menace due to corruption and bribery in the sector [22]. Similarly, Gopalakrishnan [2] indicated that, human factors such as overspeeding, drunk-driving, driver fatigue were factors that significantly contributed to RTAs. The study further indicated that, road conditons and vehicular conditons also play a contributory role in road traffic accidents. Also deaths resulting from raod traffic accidents were mostly caused by airway blockage and this may take the lives of victims in less than four(4) munites. As such, proper first aid within the first one hour after accidents may save lives [2].

Even though road safety advocacy is the mandate of the National Road Safety Authority, once the accident occurs, the patient becomes the responsibility of Ghana Health Service. As such, health policies must include RTAs looking at the increasing threats RTAs poses on the Ghana Health System. Unfortunately, the Ghana Health Service has made very little contribution in the advocacy and education of road users in Ghana. Health promotion policies have ultimately neglected human factors such as over-speeding, overtaking, drunk driving that contributes to the accidents. National Policies on road safety have been crafted to limit the issue of advocacy to the National Road Safety Authority without incorporating the Ghana Health Service. Even though the government has recently provided ambulances to provide pre-hospital care in the country, the effort requires further boost to provide appropriate pre-hospital care, rehabilitation, data collection on RTAs, implementation, and evaluation of road safety policies. The study also observed that, Ho Municipal has recently been recording the highest incidence of RTAs, also more males are involved in road traffic accidents than females. Motorcycles commit more RTAs than any other vehicles. These will certainly burden healthcare facilities in Ho and Hohoe municipalities and pose a threat to the public health of the people if urgent measures are not taken to curb the RTA menace.

## Conclusions

For all road traffic accidents that occurred in the Ho and Hohoe Municipalities in the period of study, motorcycles (three-wheeler, two-wheeler pillion, two-wheeler rider) contributed more than half of all RTA cases. All accidents involving bicycles, pedestrians, cars, motorcycles resulted in head injuries, abdomino-pelvic injury, spine injury and injury of extremity. More than half of all the respondents who had accidents with motorcycles and cars suffered head injuries. Almost all the RTA victims were working before they had their accidents. Less than half of the victims suffered a disability. However, more than half were able to return to work and few of them had lost their jobs because of the accident. The average cost of care in formal health facilities per RTA victim was found to be almost GHC1000 (US$ 167).

## Limitations

Recall bias was potentially a challenge since some of the RTA victims interviewed dates their accidents as far as 2020. However, interviewers were trained to guide RTA victims to be objective as much as possible. Pictorial diagrams were presented to victims to guide them to recollect the nature of injuries they sustained. This study employed census in three hospital facilities in the study area to recruit study participants, as such RTA cases who did not report to the hospital facilities may not be considered in the study.

## References

[1] World Health Organization. (2018). *Global status report on road safety 2018*: *Summary* (No. WHO/NMH/NVI/18.20).

[2] Gopalakrishnan S. (2012). A Public Health Perspective of Road Traffic Accidents. *Journal of Family Medicine and Primary Care*, 1(2), 144. doi: 10.4103/2249-4863.104987 24479025 PMC3893966

[3] Chen S., Kuhn M., Prettner K., & Bloom D. E. (2019). The global macroeconomic burden of road injuries: estimates and projections for 166 countries. *The Lancet Planetary Health*, 3(9), e390–e398. doi: 10.1016/S2542-5196(19)30170-6 31538624

[4] Institute for Health Metrics and Evaluation. (2019).Global Health Data Exchange. https://ghdx.healthdata.org.

[5] Islam R., Ali Khan M., Deb Nath K., Hossain M., Mustagir G., & Taneepanichskul S. (2018). Determinants of road traffic injury at Khulna division in Bangladesh: A cross sectional study of road traffic incidents [version 1; peer review: 2 approved with reservations]. *F1000Research*, 7(May), 1–16. 10.12688/F1000RESEARCH.15330.1.

[6] Shrestha V. L., Bhatta D. N., Shrestha K. M., GC K. B., & Paudel S. (2017). Factors and Pattern of Injuries Associated with Road Traffic Accidents in Hilly District of Nepal. *Journal of Biosciences and Medicines*, 05(12), 88–100. 10.4236/jbm.2017.512010.

[7] Siaw N. A., Duodu E., & Sarkodie S. K. (2013). Trends in Road Traffic Accidents in Ghana; Implications for Improving Road User Safety. *International Journal of Humanities and Social Science Invention ISSN (Online*, 2(11), 2319–7722. www.ijhssi.org.

[8] Ackaah W., & Afukaar F. K. (2010). Prevalence of helmet use among motorcycle users in Tamale Metropolis, Ghana: an observational study. Traffic injury prevention, 11(5), 522–525. doi: 10.1080/15389588.2010.489198 20872309

[9] Oteng-Ababio M., & Agyemang E. (2012). Virtue out of necessity? Urbanisation, urban growth and Okada services in Accra, Ghana. Journal of Geography and Geology, 4(1), 148–162.

[10] Dapilah F., Guba B. Y., & Owusu-Sekyere E. (2017). Motorcyclist characteristics and traffic behaviour in urban Northern Ghana: Implications for road traffic accidents. *Journal of Transport & Health*, 4, 237–245.

[11] Konlan K. D., Doat A. R., Mohammed I., Amoah R. M., Saah J. A., Konlan K. D., et al. (2020). Prevalence and Pattern of Road Traffic Accidents among Commercial Motorcyclists in the Central Tongu District, Ghana. *Scientific World Journal*, 2020. 10.1155/2020/9493718. PMC7285403 32565754

[12] Morgan A. K., Ibrahim R., Owusu A. F. S., Awafo B. A., Quartey T., Aziire M. A., et al. (2023). Prevalence and associated factors of school re-entry among teenage mothers in Ghana’s Volta Region: a cross-sectional survey. *International Journal of Adolescence and Youth*, 28(1), 2242476. 10.1080/02673843.2023.2242476.

[13] Mohammed A., Haadi A. R., & Deen A. J. (2015). Identification of risk factors involved in road accidents in Ghana. A case study of the Techiman municipality. Int J Stat Appl2015, 1, 5–14.

[14] Kudebong M., Wurapa F., Nonvignon J., Norman I., Awoonor-Williams J. K., & Aikins M. (2011). Economic burden of motorcycle accidents in Northern Ghana. Ghana medical journal, 45(4). 22359418 PMC3283097

[15] Chalya P. L., Mabula J. B., Dass R. M., Mbelenge N., & Ngayomela I. H. (2012). Injury characteristics and outcome of road traffic crash victims at Bugando Medical Centre in Northwestern Tanzania. *Journal of Trauma Management & Outcomes*, 6(1), 1. doi: 10.1186/1752-2897-6-1 22321248 PMC3292995

[16] Kumar S., Mahima, Srivastava D. K., Kharya P., Sachan N, & Kiran K. (2020). Analysis of risk factors contributing to road traffic accidents in a tertiary care hospital. A hospital based cross-sectional study. *Chinese Journal of Traumatology—English Edition*, 23(3), 159–162. 10.1016/j.cjtee.2020.04.005. PMC7296353 32381399

[17] Siddaramanna T. C., & Kumar D. R. (2014). Retrospective study of pattern of external injuries in road traffic accidents. *International Journal of Biomedical and Advance Research*, 5(09), 451–453.

[18] Slong D., & Devi T. M. (2013). Pattern of Head Injuries in Road Traffic Accidents in Imphal. *Medico-Legal Update*, 13(1), 6.

[19] Mittal S., Tayal I., Garg S., & Gupta N. Pattern of common injuries in road traffic accidents. *Brain*, 287, 56–16.

[20] Kumar P. R.(2013) A Study on Pattern of Head Injuries in Two Wheeler Road Traffic Accidents. International Journal of Health Sciences and Research www.ijhsr.org ISSN: 2249-9571.

[21] Juillard C., Labinjo M., Kobusingye O., & Hyder A. A. (2010). Socioeconomic impact of road traffic injuries in west Africa: Exploratory data from Nigeria. *Injury Prevention*, 16(6), 389–392. doi: 10.1136/ip.2009.025825 20805620

[22] Coleman A. (2014). Road Traffic Accidents in Ghana: A Public Health Concern, and a Call for Action in Ghana, (and the Sub-Region). *Open Journal of Preventive Medicine*, 04(11), 822–828. 10.4236/ojpm.2014.411092.

